# A clinical study of topical treatment for thyroid-associated ophthalmopathy with dry eye syndrome

**DOI:** 10.1186/s12886-023-02805-8

**Published:** 2023-02-20

**Authors:** Rou Sun, Muyue Yang, Chenyi Lin, Yu Wu, Jing Sun, Huifang Zhou

**Affiliations:** Department of Ophthalmology, Shanghai Key Laboratory of Orbital Diseases and Ocular Oncology, Ninth People’s Hospital, Shanghai Jiao Tong University School of Medicine, Shanghai, 200011 China

**Keywords:** Thyroid-associated ophthalmopathy, Dry eye syndrome, Vitamin A palmitate eye gel

## Abstract

**Introduction:**

Clinically, thyroid-associated ophthalmopathy (TAO) patients were suffered from dry eye syndrome. Only a few relevant studies were about this topic. Our study was determined to provide high-level evidence for the treatment of TAO with dry eye syndrome.

**Purpose:**

To compare the clinical effects of vitamin A palmitate eye gel and sodium hyaluronate eye drop forTAO patients with dry eye syndrome.

**Methods:**

The study was conducted in the Ophthalmology Department of the Ninth People’s Hospital Affiliated with the Medical College of Shanghai Jiao Tong University from May to October 2020. A total of 80 mild or moderate-to-severe TAO patients with dry eye syndrome were randomly divided into two groups. The disease stages of all subjects were inactive. Patients in group A were treated with vitamin A palmitate eye gel three times/day for one month and sodium hyaluronate eye drop in group B. The index including break-up time (BUT) and Schirmer I test (ST), corneal fluorescence staining (FL), ocular surface disease index (OSDI), and adverse reactions were recorded by the same clinician at baseline and 1 month after treatment. The data were analyzed by SPSS 24.0.

**Results:**

Finally, 65 subjects completed the treatment. The average age of the patients in Group A was 38.1 ± 11.4 years, and that in Group B was 37.26 ± 10.67 years. 82% of the subjects in group A were female and 74% in group B. There was no significant difference between the two groups at baseline, including the value of ST, BUT, OSDI, and FL grade. After the treatment, the effective rate was 91.2% in group A, of which the value of BUT and FL grade was significantly improved (*P* < 0.001). The effective rate in group B was 67.7%, of which the value of OSDI score and FL grade was significantly improved (*P* = 0.002). In addition, the BUT value of group A was significantly longer than that of group B (*P* = 0.009).

**Conclusion:**

InTAO patients with dry eye syndrome, vitamin A palmitate gel and sodium hyaluronate eye drop improved the dry eye and promoted corneal epithelial repair. Vitamin A palmitate gel improves the stability of tear film, while sodium hyaluronate eye drop improves patients’ subjective discomfort.

**Supplementary Information:**

The online version contains supplementary material available at 10.1186/s12886-023-02805-8.

## Introduction

Thyroid-associated ophthalmopathy (TAO), which also called Graves’ ophthalmopathy (GO) or Thyroid eye disease (TED), is an autoimmune disease that usually occurs in patients with Graves’ disease or autoimmune thyroiditis [1–6].

TAO patients suffer from exophthalmos, eyelid retraction and eye movement disorder [7]. Dry eye syndrome is one of the most common reasons for eye discomfort in patients with TAO, with a high incidence of 65%-85% [8–10]. Possible causes of dry eye are as follows. Firstly, the inflammatory reaction of the lacrimal gland, the increase in incomplete blink rate and the loss of the meibomian gland can influence the secretion of tear and alter the tear composition [11–14]. In the case of the lacrimal gland of TAO patients, local lymphocyte infiltration may increase its volume, altering the tear composition [15]. The levels of inflammatory proteins and cytokines increase in TAO patients’ tears [16–18]. Secondly, exophthalmos and eyelid retraction increases the ocular surface exposure, resulting in tear evaporation and elevated osmotic pressure [19]. In addition, Stellwag’s sign and aggravated eye movement disorder can further damage the cornea and conjunctiva [15]. TAO patients had lower superficial epithelial cell density in the superior bulbar conjunctiva, increased Langerhans cell density, and reduced goblet cell density. A high degree of squamous metaplasia was revealed in the conjunctival epithelium of TAO patients [14]. Corneal sensitivity is also affected. TAO patients had fewer epithelial corneal cells, lower sub-basal nerve density, and more stromal corneal cells compared to the normal subjects [20]. Therefore, TAO leads to dry eye syndrome with complex pathogenesis. Currently, only a few relevant studies are available on the treatment of TAO with dry eye syndrome, albeit controversial and not a distinct standardized treatment regimen [21]. Therefore, this study enrolled TAO patients with dry eye syndrome and randomly categorized them into Group A and Group B; vitamin A palmitate eye gel or sodium hyaluronate eye drop was applied three times a day independently to evaluate the efficacy and safety of the treatment.

## Materials and methods

This was a prospective, randomized clinical study. A total of 80 mild or moderate-to-severe TAO patients (80 eyes) with dry eye syndrome were selected from the Ophthalmology Department of the Ninth People’s Hospital Affiliated to the Medical College of Shanghai Jiao Tong University from May to October 2020. The disease stages of all subjects were inactive. The severely affected eye in each patient was considered in the study. The inclusion criteria were as follows: ① Diagnosed as mild or moderate-to-severe TAO and the disease stage was inactive.; ② Diagnosed as dry eye syndrome. The exclusion criteria were as follows: ① Suffering from other ophthalmic diseases affecting the ocular surface; ② Suffering from autoimmune diseases, such as systemic lupus erythematosus, rheumatoid arthritis, and Sjögren’s syndrome; ③ Previous ocular trauma or surgery; ④ Extremely severe TAO; ⑤ Allergic to drug ingredients. The content of this study was approved by the medical ethics committee of our hospital (approval No. sh9h-2019-t343-2, approved on March 18th, 2020) and registered in the Chinse clinical trial registry (Registration no. chictr2000033874, approved on 15/06/2020). Informed consent was signed by all subjects.

TAO was diagnosed according to the Bartley standard [22]. If eyelid retraction was detected, one of the following three signs could be diagnosed as TAO: ① Exophthalmos; ② History of thyroid dysfunction; ③ Extraocular muscle involvement. If eyelid retraction was not found, all the three conditions should be fulfilled: ① History of thyroid dysfunction; ② Combined with exophthalmos, extraocular muscle involvement, or visual dysfunction; ③ Excluding eyelid retraction, exophthalmos, and eye movement disorder caused by other eye diseases.

Mild TAO was defined as patients who could not meet the diagnostic criteria of moderate-to-severe TAO. Moderate-to-severe TAO was defined if a patient had no visual impairment but at least two of the following manifestations: ① Eyelid retraction: upper lid margin to reflex distance (MRD-1) was > 5 mm or a lower lid margin to reflex distance (MRD-2) was > 6 mm. ② Moderate-to-severe soft tissue damage (one of the following: moderate-to-severe eyelid swelling or moderate-to-severe conjunctival congestion); ③ At least 3 mm over the normal value of proptosis (Hertel exophthalmos of any eye ≥ 17 mm); ④ Stable or intermittent diplopia.

Extremely severe TAO was defined as patients with compressive optic neuropathy (DON) and/or severe exposure keratopathy.

The following was the judgment criteria for active and inactive TAO [23]. The clinical activity score (CAS) was used to evaluate active and inactive TAO. The scale comprised seven items, including eyelid edema, eyelid congestion, conjunctival congestion, conjunctival edema, lacrimal caruncle redness and swelling, spontaneous retrobulbar pain, and pain during gaze or eye movement. Each of the items was scored 1 point, < 3 points as inactive period, and ≥ 3 points as the active period.

TAO with dry eye syndrome was defined as fulfilling any of the following items based on the diagnosis of TAO [24]: ① having one of the subjective symptoms (visual fatigue, discomfort, foreign body, dryness, burning, and visual acuity fluctuation) and Schirmer I test (ST) ≤ 10 mm; ② One of the subjective symptoms and break-up time (BUT) ≤ 10 s; ③ Positive fluorescein staining of keratoconjunctiva.

### The chemical composition

0.1% sodium hyaluronate eye drop: Sodium hyaluronate of 2 million to 3 million Daltons is the main component. The concentration is 0.1% and it does not contain preservatives. Inherent viscosity is 2.4–3.2m^3^/kg.

Vitamin A palmitate eye gel: Vitamin A palmitate of 524.87 Daltons is the main component. The concentration is 0.8 mg/g and it contains preservatives. Inherent viscosity is 3.2–4.2m^3^/kg.

### Sample Size

In the pre-experiment, 10 patients who applied Vitamin A palmitate eye gel got an effective rate of 60% in improving dry eye while 10 who applied 0.1% sodium hyaluronate eye drop got 30%. We let the inspection level α = 0.049, inspection efficiency 1- β = 0.80. Considering the possible loss of follow-up rate as 20%, the total sample size of this study was 80 and 40 in each group.

In this study, 80 patients (80 eyes) were randomly divided into Group A and Group B by the envelope method. Vitamin A palmitate eye gel was applied in Group A three times/day, one drop each time. Group B was given 0.1% sodium hyaluronate eye drop three times/day, one drop each time. The effect was evaluated 1 month after drug administration. During the treatment, other topical eye drugs were not allowed.

The following data of subjects were collected at baseline and after 1 month of treatment. In consideration of the actual situation, clinical data were collected three days before or after one month was allowed. Generally, gel or eye drops were not used on the day of measurement, and the measurement time was at the same time of the day at baseline or post treatment.

### ST

The inverted end of the long tear detection filter paper was placed about 5 mm into the outer 1/3^rd^ of the subject's lower eyelid conjunctival sac without ocular surface anesthesia, and the subject was asked to close his eyes gently. After 5 min, the filter paper was taken down, and the ST value was recorded.

### BUT

Corneal topography (Keratograph 5 M, type 77,000, Germany) was applied. After the subject placed his head in the specified area, the BUT function option of the software, OCULUS Keratograph, was selected. The subject was instructed to look forward without blinking. The value of BUT was obtained after the examination.

### Fluorescence staining (FL)

The range of fluorescein staining was observed under the cobalt blue light of the slit lamp microscope after placing the 2% sodium fluorescein staining test paper into the subject’s conjunctival sac of the lower eyelid. The cornea was divided into four quadrants: supratemporal, infratemporal, supranasal, and infranasal. The score was evaluated in every quadrant. The scoring criteria were as follows: 0 for no coloring, 1 for scattered punctate coloring, 2 for diffuse punctate coloring, and 3 for massive coloring. The sum of the scores in each quadrant constituted the corneal FL score with a total of 0–12 points.

### Ocular surface disease index (OSDI)

The subjects were required to answer 12 questions, and each question was scored 0–4 points [25]. Nonetheless, the subject could refuse to answer some of the questions. The total score was multiplied by 25 and then divided by the number of questions answered to obtain the final score.

### Effective rate

The effective rate was if any of the following three conditions were satisfied. ① The value of ST was improved by 2 mm after treatment; ② The value of BUT was improved by 2 s after treatment; ③ The FL was decreased by 1 point after treatment [26].

### Statistical analysis

The statistician generated a random sequence, and the clinician who made the evaluation was blind to the grouping. The data were statistically analyzed using SPSS 24.0. The continuous data obeying the normal distribution were expressed as mean ± standard deviation, and non-normal data were represented as median (interquartile range). The paired sample t-test or Wilcoxon signed-rank test was used for the comparison between the two groups of paired samples, and the independent sample t-test or Mann–Whitney U test was used for the comparison between the two groups of independent samples. Categorical data were described as the number of cases (percentage), and the chi-square test was used for intergroup comparison. *P* < 0.05 indicated statistically significant difference.

## Results

**Fig. 1:**
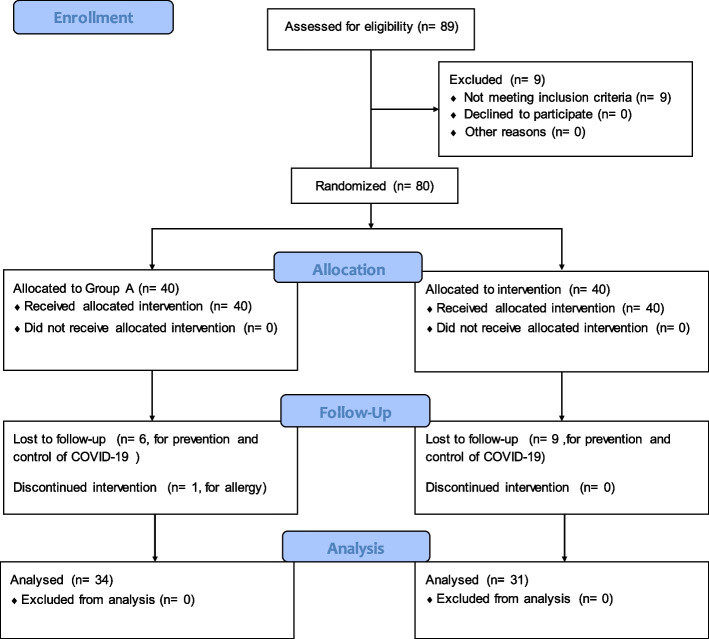
Study flow chart

A total of 80 mild or moderate-to-severe TAO patients (80 eyes) with dry eye syndrome were enrolled in this study. The disease stages of all subjects were inactive. Subsequently, 6 patients in Group A and 9 patients in Group B were lost to follow-up (Fig. 1). The other patients completed the treatment according to the plan. The average age of the patients in Group A was 38.10 ± 11.40 years, and that in Group B was 37.26 ± 10.67 years. No significant difference was observed between the two groups in age, gender, clinical activity score (CAS), exophthalmos, MRD-1, MRD-2, visual acuity, intraocular pressure, the values of ST, BUT, and OSDI, and FL grade (*P* > 0.05, Table 1).

**Table 1:** Baseline conditions of the two groups before treatment

Indicator	Group A (*n* = 34)	Group B (*n* = 31)	*P*-value
Age (years)	37.26 ± 10.67	38.10 ± 11.40	0.760
Sex—Female	28	23	0.309
Male	6	8	
CAS	1.35 ± 1.04	1.42 ± 1.21	0.989
Proptosis (mm)	18.69 ± 2.62	18.19 ± 2.77	0.460
MRD-1 (mm)	5.06 ± 1.94	4.97 ± 1.64	0.839
MRD-2 (mm)	5.68 ± 1.27	5.52 ± 1.00	0.576
Vision acuity	0.93 ± 0.14	0.95 ± 0.09	0.623
IOP (mmHg)	19.15 ± 3.02	18.71 ± 2.51	0.529
ST (mm)	8.31 ± 6.28	6.40 ± 5.85	0.174
BUT (s)	6.17 ± 4.43	6.28 ± 3.85	0.434
OSDI	19.6 (9.30–43.13)	21.87 (8.33–43.18)	0.960
FL	2 (1–5)	2 (1–2)	0.255

*CAS* clinical activity score, *MRD-1* upper lid margin to reflex distance, *MRD-2* lower lid margin to reflex distance, *IOP* intraocular pressure, *BUT* break-up time, *ST* Schirmer I test, *FL* fluorescence staining, *OSDI* ocular surface disease index

In Group A, ST increased from 8.61 ± 6.34 mm to 9.13 ± 7.1 mm, and the score of the OSDI scale improved from 19.60 (9.30–43.13) to 14.58 (6.67–31.25) (*P* > 0.05). In addition, BUT increased from 6.38 ± 4.48 s to 9.84 ± 3.76 s (*P* < 0.001) and the FL grade improved from 2 (1–5) to 1 (0–2) (*P* < 0.001). After 1-month of treatment, the stability of tear film improved and the corneal epithelial injury was repaired.

In Group B, ST increased from 5.78 ± 4.81 mm to 6.37 ± 5.23 mm and BUT increased from 6.28 ± 3.85 s to 7.17 ± 4.08 s, with no significant difference (*P* > 0.05). The score of OSDI scale improved from 21.87 (8.33–43.18) to 9.09 (5.55–25) (*P* = 0.002), and the FL grade improved from 2 (1–2) to 1 (0–2) (*P* = 0.002). Subsequently, the subjective discomfort of the patients improved, and the corneal epithelial injury was repaired. Also, a significant difference was detected in BUT after treatment (*P* = 0.009), but no difference in ST, OSDI, and FL grade between the two groups (Tables 2 and 3).

**Table 2:** Comparison of dry eye indexes between the two groups after treatment

Indicator	Group A	Group B	*P*-value
ST (mm)	9.13 ± 7.10	6.37 ± 5.23	0.088
BUT (s)	9.84 ± 3.76	7.17 ± 4.08	0.009
OSDI	14.58 (6.67–31.25)	9.09 (5.55–25.00)	0.580
FL	1 (0–2)	1 (0–2)	1.000

*BUT* break-up time, *ST* Schirmer I test, *FL* fluorescence staining, *OSDI* ocular surface disease index

**Table 3:** Comparison of dry eye indexes in two groupsbefore and after treatment

		Before	After	*P*-value
Group A	ST (mm)	8.61 ± 6.34	9.13 ± 7.10	0.402
	BUT (s)	6.38 ± 4.48	9.84 ± 3.76	< 0.001
	OSDI	19.6 (9.3–43.13)	14.58 (6.67–31.25)	0.061
	FL	2 (1–5)	1 (0–2)	< 0.001
Group B	ST (mm)	5.78 ± 4.81	6.37 ± 5.23	0.440
	BUT (s)	6.28 ± 3.85	7.17 ± 4.08	0.217
	OSDI	21.87 (8.33–43.18)	9.09(5.55–25.00)	0.002
	FL	2 (1–2)	1 (0–2)	0.002

*BUT* break-up time, *ST* Schirmer I test, *FL* fluorescence staining, *OSDI* ocular surface disease index

Among the subjects, 17 patients had an ST increase > 2 mm, 30 had a BUT enhancement > 2 mm, and 36 had an FL grade improvement > 1 point. The total effective rate was 80.0% (52/65). In group A, 10 patients had an ST increase > 2 mm, 21 had a BUT enhancement > 2 mm, and 21 had a FL grade improvement > 1 point, with an effective rate of 91.2% (31/34). In group B, 7 patients had an ST increase > 2 mm, 9 had a BUT enhancement > 2 mm, and 13 had an FL grade improvement > 1 point, with an effective rate of 67.7% (21/31). Strikingly, a significant difference was observed between the two groups (*P* = 0.018).

In terms of safety, only one case of drug allergy occurred in Group A, and after stopping the application of the eye gel, conjunctival hyperemia disappeared in 1 day without any complications.

## Discussion

Dry eye syndrome is an important factor that causes eye discomfort in TAO patients clinically, which is closely related to the increase of tear evaporation and osmotic pressure caused by the increase of ocular surface exposure, the change of tear composition caused by eye inflammation, and eye movement disorders. However, the treatment for TAO with dry eye syndrome has not been standardized, and there are few articles on related issues. Therefore, this article aimed to provide high-quality clinical evidence for the treatment of TAO patients with dry eye syndrome.

The first problem was the selection of research objects. The ocular manifestations of patients with extremely severe TAO or with active TAO are still changing, so it is difficult to distinguish whether the effect of drug treatment is affected by the development of the disease. Therefore, we choose patients with mild or moderate-to-severe TAO patients as subjects and the disease stages of all subjects were inactive. At baseline, we evaluated the patients’ age and gender. In our study, the average age of the subjects was slightly lower than the results of some previous study [27, 28]. Dolman et al. demonstrated that the risk factors of DON included advancing age and male gender, which might explain this problem [29]. There was no difference in age and exophthalmos between the two groups, and the results were consistent with those mentioned in other studies [28, 30–32]. In addition, the value of MRD, CAS, visual acuity and IOP at baseline all showed no difference between the two groups.

ST and BUT are objective indexes to evaluate the dry eye, which reflect the stability of tear film and basic tear secretion. These two indicators have decreased in varying degrees in TAO patients with dry eye syndrome because of the increase of exposure factors, the occurrence of inflammation and the reduction of tear secretion. The ST of Group A increased from 8.61 ± 6.34 mm to 9.13 ± 7.1 mm (*P* = 0.402) and BUT increased from 6.38 ± 4.48 s to 9.84 ± 3.76 s (*P* < 0.001) after treatment, while ST of Group B increased from 5.78 ± 4.81 mm to 6.37 ± 5.23 mm and BUT increased from 6.28 ± 3.85 s to 7.17 ± 4.08 s (*P* > 0.05). It indicated that vitamin A palmitate eye gel was superior to sodium hyaluronate eye drop in improving the tear film stability of TAO patients with dry eye syndrome, which could take effect in 1 month. Sodium hyaluronate is a commonly used artificial tear drug in the clinic. It has good viscoelasticity and hygroscopicity, and the carboxyl and hydroxyl groups in the molecular structure produce hydrogen bonds with water with significant water retention. For other dry eye syndromes, such as those appearing after cataract surgery, sodium hyaluronate eye drop improves the patients' symptoms and signs [33–35]. Vitamin A palmitate gel maintains the stability of the tear film, increases the density of goblet cells, and promotes the secretion of goblet cells and lacrimal gland cells [21, 36–38]. It also promotes intercellular connections and reverses keratosis of corneal epithelial cells. Vitamin A palmitate eye gel reduces the incidence of dry eye syndrome after strabismus surgery and increases the density of goblet cells, improving dry eye caused by prostaglandin analog [39, 40]. Altiparmak et al. demonstrated found that 6 months after using artificial tear drops, ST of TAO patients with dry eye syndrome increased from 3.73 ± 1.85 mm to 10.73 ± 5.71 mm and BUT increased from 6.21 ± 1.73 s to 12.9 ± 3.96 s [41]. However, applying artificial tears and cyclosporine A increased ST from 3.32 ± 1.99 mm to 6.68 ± 4.42 mm and increased BUT from 6.36 ± 2.02 s to 10.92 ± 0.76 s. We found that the improvement of ST and BUT in our study was lesser than that reported by Altiparmak et al.. These phenomena might be caused by the short period of treatment time. We speculated that prolonged medication could achieve significant results. Although vitamin A palmitate eye gel can increase the number of goblet cells, it was not sufficient for a significant difference in ST in a short-term observation of 1 month. The inflammatory reaction of the lacrimal gland in TAO patients reduced the secretion of tears, which might not be improved by vitamin A palmitate eye gel or sodium hyaluronate eye drop. Also, the exposure factors were not resolved, which might explain why ST did not increase significantly in both groups.

FL examination can be used to assess the degree of corneal epithelial injury. Due to the increase of ocular surface exposure and exophthalmos, the FL grade of TAO patients with dry eye syndrome would increase as the baseline data showed. A past study showed that the topical use of Rebamipide for 1 month improves the upper limbal keratoconjunctivitis of TAO patients and repairs the corneal injury [42]. In our study, according to corneal fluorescein staining, applying vitamin A palmitate eye gel or sodium hyaluronate eye drop significantly reduced the FL grade, promoting the repair of corneal epithelial cells. However, the number of patients whose FL grade improved > 1 point after treatment in Group A was 21 and 9 in Group B. For patients with dry eye syndrome, sodium hyaluronate eye drop improves the FL grade. The synthesis of cytokine receptors and glycoproteins is promoted by vitamin A palmitate gel that can shorten the epithelial healing time and improve dry eye [39, 43, 44]. A previous study demonstrated that vitamin A palmitate eye gel combined with sodium hyaluronate eye drop can relieve the symptoms of dry eye patients and also significantly reduce FL grade and the level of inflammatory factors in tears [21]. This phenomenon that FL grade improved more in group A after treatment could be attributed to the gelatinous nature of vitamin A palmitate eye gel, prolonging the retention time in the conjunctival sac. This, in turn, promoted the repair of corneal epithelium.

The OSDI scale evaluates the subjective feeling of the patients [45]. TAO patients have reduced tear secretion, decreased tear film stability, damaged corneal epithelium, and can feel discomfort, such as foreign body, fatigue, burning, and decreased vision, which will increase their OSDI score [10, 22]. Gürdal et al. demonstrated that after 2 months of topical cyclosporine A treatment, OSDI score decreased significantly (*P* = 0.001) [46, 47]. In our study, the OSDI score of Group B was significantly lower after treatment than before the treatment, suggesting that the patient's subjective symptoms were improved. Although the OSDI score of Group A improved, no significant difference was detected after treatment compared to that at baseline. Sodium hyaluronate is conducive to improving the congestion of bulbar conjunctiva and reducing the local inflammatory reaction and negative psychological emotion of patients with high safety [48, 49]. Retinol palmitate ophthalmic solution has also been proven beneficial to the objective signs and subjective symptoms of dry eye syndrome [50]. Though both groups had an improved OSDI score, significant differences existed only in group B. This might be due to a slight pain in the eyes of some patients after using vitamin A palmitate eye gel, which affected the feelings of patients. The gel might influence the visual acuity of subjects after application. Herein, we recorded one case of drug allergy in Group A, which was recovered after drug withdrawal.

Overall, the efficiency of vitamin A palmitate eye gel on TAO with dry eye syndrome was significantly higher than that of sodium hyaluronate eye drop. Both improved corneal epithelial injury and index for evaluating the dry eye. Therefore, these two drugs could be utilized clinically to improve the dry eye syndrome of TAO patients. Nevertheless, the present study has some deficiencies. It is a single-center study with a small sample size and short observation time. TAO patients with different disease stages or grading may have varied responses to the topical eye drops. In addition, 15 subjects in this study missed their follow-up due to the COVID-19 pandemic. However, this study still has high credibility because the initial sample size included a lost visit rate of only 20%. Further studies should be focused on other drugs such as sodium hyaluronate eye drops with higher concentration and the superimposed use of drugs to explore the local treatment scheme of TAO with dry eye syndrome. In the past studies, the description of the role of cyclosporine in TAO patients with dry eye syndrome had the opposite conclusion [41, 46, 47]. Clinical data, such as the tear osmolarity measurement, the number of goblet cells, corneal sensitivity, and corneal nerve fiber density, biomarkers, can be added to future studies, providing evidence for the standard treatment for TAO with dry eye syndrome [51, 52].

## Conclusion

Both 0.1% sodium hyaluronate eye drop and vitamin A palmitate gel can be used in the treatment of TAO with dry eye syndrome. Both drugs promote corneal epithelial repair; vitamin A palmitate eye gel can improve tear film stability, and sodium hyaluronate eye drop can improve the patients’ subjective symptoms. Thus, vitamin A palmitate eye gel is more effective than 0.1% sodium hyaluronate eye drop for treating TAO with dry eye syndrome.

## Supplementary Information

**Additional file 1.**

## Data Availability

All data generated or analyzed during this study are included in this published article.
