# Supplementary material for: A clinical study of topical treatment for thyroid-associated ophthalmopathy with dry eye syndrome
Source: BMC Ophthalmol. 2023 Feb 20;23:72. doi: 10.1186/s12886-023-02805-8 (PMC9940084; doi:10.1186/s12886-023-02805-8)
Supplement: Supplementary file 1 — Additional file 1. [file 12886_2023_2805_MOESM1_ESM.docx]

Trial Protocol

Project summary

Up to 65% to 85% TAO patients suffer from dry eye^[1]^. However, at present, there are few relevant studies on the treatment of TAO with dry eye, and no clear standardized treatment scheme^[2]^. Therefore, this study enrolled TAO patients with dry eye and randomly separated them into the experimental group (Group A) and the control group (Group B). Then we evaluated the efficacy and safety of vitamin A palmitate eye gel compared with sodium hyaluronate eye drops in the treatment of TAO with dry eye. Our study was conducted in the Ophthalmology Department of the Ninth People’s Hospital Affiliated with the Medical College of Shanghai Jiao Tong University. 80 mild or moderate-to-severe TAO patients with dry eye were divided into a control group and an experimental group with 40 eyes each. Patients in the experimental group were treated with vitamin A palmitate eye gel three times/day for one month and sodium hyaluronate eye drop in the control group. The purpose was to evaluate the clinical effect of vitamin A palmitate eye gel and sodium hyaluronate eye drop for thyroid-associated ophthalmopathy (TAO) with dry eye syndrome, and to provide evidence-based medical evidence for the development of treatment for ocular surface problems of TAO patients.

General information

Protocol title: A clinical study of topical treatment for thyroid-associated ophthalmopathy with dry eye syndrome

Department of Ophthalmology, Ninth People’s Hospital, Shanghai Jiao Tong University School of Medicine

Address: No. 500, Quxi Road, Huangpu District, Shanghai

Shanghai Key Laboratory of Orbital Diseases and Ocular Oncology, Shanghai 200011, China

Address: No. 833, Zhuangju Road, Huangpu District, Shanghai

Tel: 15221670562

Project management

Study design: Jing Sun, Huifang Zhou;

Implementation of the study: Rou Sun, Muyue Yang, Chenyi Lin, Yu Wu;

Manuscript writing: Rou Sun, Muyue Yang;

Huifang Zhou: Chief physician, Ph D

Jing Sun: Deputy chief physician, Ph D

Rou Sun, Muyue Yang, Chenyi Lin, Yu Wu: MD

Date: November 25th, 2019

Expected duration of the project

Collecting data: From May, 2020 to March, 2021

Statistical analysis: From April, 2021 to June, 2021

Manuscript writing: From June, 2021 to September, 2021

Ethics

The content of this study was approved by the medical ethics committee of our hospital (approval No. sh9h-2019-t343-2, approved on March 18th, 2020)

Rationale & background information

Thyroid-associated ophthalmopathy (TAO) is an orbitopathy with highest incidence rate, which often takes place in patients suffering from Graves’ disease with hyperthyroidism or a history of hyperthyroidism^[3,4]^. It can also occur in people with autoimmune thyroiditis and with normal or decreased thyroid function. TAO is an autoimmune disease whose pathogenesis is not clear. It may be caused by orbital inflammatory reaction induced by cross reactive antigen between orbital tissue and thyroid tissue^[5]^. TAO involves eyelids, extraocular muscles, orbital fat and lacrimal glands and can lead to symptoms such as exophthalmos, eyelid swelling, eyelid retraction, diplopia, photophobia and tears, vision loss, strabismus and eye movement disorder^[6]^. Exophthalmos and eyelid retraction lead to the increase of ocular surface exposure, resulting in more tear evaporation and elevated osmotic pressure^[7]^. In addition, the inflammatory reaction of lacrimal gland, the increase of incomplete blink rate and the loss of meibomian gland can lead to the change of tear composition and the decrease of aqueous tear as well as goblet cells^[8-10]^. The decrease of tear stability, corneal sensitivity and the aggravation of eye movement disorder can further cause the damage of cornea and conjunctiva.

Vitamin A palmitate gel can maintain the stability of tear film, increase the density of goblet cell and promote secretion of goblet cells and lacrimal gland cells^[11]^. It promotes the establishment of intercellular connections and reverses keratosis of corneal epithelial cells. The synthesis of cytokine receptors and glycoproteins can be promoted by vitamin A palmitate gel, which can shorten the epithelial healing time and improve dry eye^[12-13]^. Sodium hyaluronate is a commonly used artificial tear drug in clinic. It has good viscoelasticity and hygroscopicity, whose carboxyl and hydroxyl groups in the molecular structure can also produce hydrogen bonds with water, having good water retention. It is conducive to improving the congestion of bulbar conjunctiva, and the value of break-up time (BUT) as well as schirmer I test (SIT) . At the same time, it can reduce the local inflammatory reaction and psychological negative emotion of patients with high safety. Therefore, this study enrolled TAO patients with dry eye and randomly separated them into the experimental group (Group A) and the control group (Group B). Then we evaluated the efficacy and safety of vitamin A palmitate eye gel compared with sodium hyaluronate eye drops in the treatment of TAO with dry eye.

References

[1] Ismailova D S, Fedorov A A, Grusha Y O. Ocular Surface Changes in Thyroid Eye Disease. Orbit. 2013;32(2):87-90.

[2] Sun R, Zhou HF, Fan XQ, Ocular surface changes in Graves' ophthalmopathy.[J] .Int J Ophthalmol, 2021, 14: 616-621.

[3] Bartalena L. Prevention of Graves’ ophthalmopathy. Best Practice & Research Clinical Endocrinology & Metabolism. 2012;26(3): 371-379.

[4] Longo D L, Smith T J, Hegedüs, et al. Graves Disease. New England Journal of Medicine. 2016;375(16):1552-1565.

[5] Wang Y, Smith T J. Current Concepts in the Molecular Pathogenesis of Thyroid-Associated Ophthalmopathy. Investigative Opthalmology & Visual Science. 2014;55(3):1735.

[6] Bartalena L, Tanda ML. Graves ’ Ophthalmopathy. New England Journal of Medicine. 2009;362(8):726-738.

[7] Jeffrey P G M D, Farris R L, Gilbard J P. Ocular surface drying and tear film osmolarity in thyroid eye disease. Acta ophthalmologica. 1983;61(1):108-116.

[8] Song RH, Wang B, Yao QM, et al. Proteomics screening of differentially expressed cytokines in tears of patients with Graves' ophthalmopathy. Endocr Metab Immune Disord Drug Targets, 2020, 20: 87-95.

[9] Jinhwan, Park, Sehyun, et al. Dry eye syndrome in thyroid eye disease patients: The role of increased incomplete blinking and Meibomian gland loss. Acta ophthalmologica. 2019;97: e800-e806.

[10] Wei Y, Chen W, Hu F, et al. In vivo confocal microscopy of bulbar conjunctiva in patients with Graves' ophthalmopathy. Journal of the Formosan Medical Association. 2013;114(10):965-972.

[11] Hiroshi T , Toshinari F , Koichi O , et al. Efficacy and safety of retinol palmitate ophthalmic solution in the treatment of dry eye: a Japanese Phase II clinical trial[J]. Drug Design Development & Therapy, 2017, 11:1871-1879.

[12] Cui X , Xiang J , Zhu W , et al. Vitamin A Palmitate and Carbomer Gel Protects the Conjunctiva of Patients With Long-term Prostaglandin Analogs Application[J]. Journal of Glaucoma, 2016, 25(6):487.

[13] Tabuchi, Nobuhito, Toshida, et al. Effect of Retinol Palmitate on Corneal and Conjunctival Mucin Gene Expression in a Rat Dry Eye Model After Injury[J]. Journal of Ocular Pharmacology & Therapeutics the Official Journal of the Association for Ocular Pharmacology & Therapeutics, 2017.

Study goals and objectives

To evaluate the clinical effect of vitamin A palmitate eye gel and sodium hyaluronate eye drop for thyroid-associated ophthalmopathy (TAO) with dry eye syndrome

Expected outcomes of the study

To provide evidence-based medical evidence for the development of treatment for ocular surface problems of TAO patients.

Study design

The type of study: A prospective, randomized clinical study (Figure 1)

The research population: Mild or moderate-to-severe TAO patients with dry eye

The expected duration of the study: Starts from May, 2020 and ends when 80 patients are recruited in the study


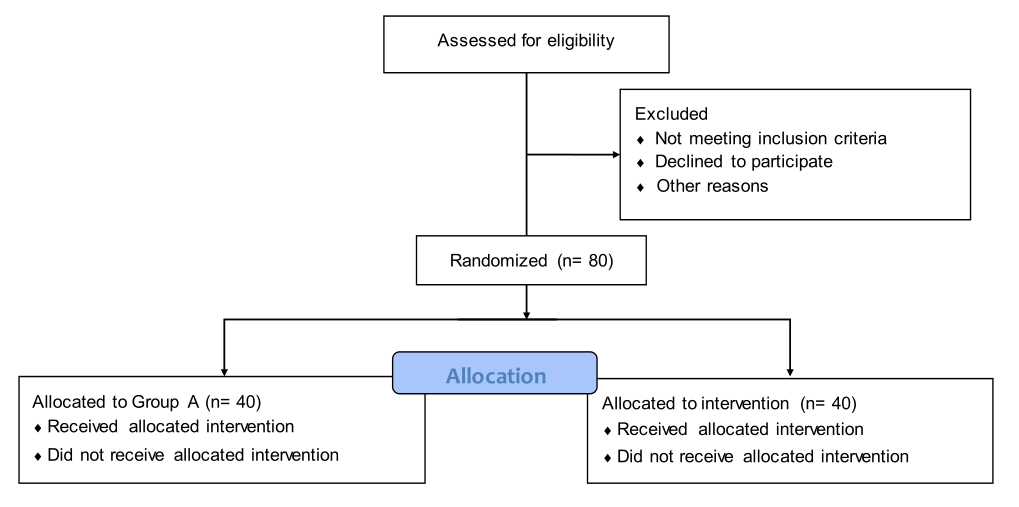


Figure1. The flow diagram of the study

Patients in experimental group were treated with vitamin A palmitate eye gel three times a day for a month, while with sodium hyaluronate eye drop in control group. The index of dry eye test, including break-up time (BUT) and schirmer I test (SIT), as well as corneal fluorescence staining (FL), ocular surface disease index (OSDI) and adverse reactions were analyzed. The data were statistically analyzed by SPSS 24.0.

Sample Size

In the pre-experiment, patients who applied Vitamin A palmitate eye gel got an effective rate of 60% while who applied 0.1% sodium hyaluronate eye drop got 30%. We let the inspection level α= 0.049, inspection efficiency 1- β= 0.80. Considering the possible loss of follow-up rate as 20%, the total sample size of this study was 80 and 40 in each group.

Inclusion criteria and Exclusion criteria

Inclusion criteria ① Diagnosed as mild or moderate-to-severe inactive TAO; ② Diagnosed as dry eye syndrome.

Exclusion criteria ① Suffering from other ophthalmic diseases affecting the ocular surface; ② Suffering from autoimmune diseases, such as systemic lupus erythematosus, rheumatoid arthritis, and Sjögren’s syndrome; ③ Previous ocular trauma or surgery; ④ Extremely severe TAO; ⑤ Allergic to drug ingredients. The content of this study was approved by the medical ethics committee of our hospital (approval No. sh9h-2019-t343-2, approved on March 18th, 2020) and registered in the Chinse clinical trial registry (Registration no. chictr2000033874, approved on June 15th, 2020). Informed consent was signed by all subjects.

TAO was diagnosed according to the Bartley standard. If eyelid retraction was detected, one of the following three signs could be diagnosed as TAO: ① Exophthalmos; ② History of thyroid dysfunction; ③ Extraocular muscle involvement. If eyelid retraction was not found, all the three conditions should be fulfilled: ① History of thyroid dysfunction; ② Combined with exophthalmos, extraocular muscle involvement, or visual dysfunction; ③ Excluding eyelid retraction, exophthalmos, and eye movement disorder caused by other eye diseases.

Mild TAO was defined as patients who could not meet the diagnostic criteria of moderate-to-severe TAO. Moderate-to-severe TAO was defined if a patient had no visual impairment but at least two of the following manifestations: ① Eyelid retraction: upper lid margin to reflex distance (MRD-1) was > 5 mm or a lower lid margin to reflex distance (MRD-2) was > 6 mm. ② Moderate-to-severe soft tissue damage (one of the following: moderate-to-severe eyelid swelling or moderate-to-severe conjunctival congestion); ③ At least 3 mm over the normal value of proptosis (Hertel exophthalmos of any eye ≥ 17mm); ④ Stable or intermittent diplopia.

Extremely severe TAO was defined as patients with compressive optic neuropathy (DON) and/or severe exposure keratopathy.

Judgment criteria for active and inactive TAO

The clinical activity score (CAS) was used to evaluate active and inactive TAO. The scale comprised seven items, including eyelid edema, eyelid congestion, conjunctival congestion, conjunctival edema, lacrimal caruncle redness and swelling, spontaneous retrobulbar pain, and pain during gaze or eye movement. Each of the items was scored 1 point, < 3 points as inactive period, and ≥ 3 points as the active period.

TAO with dry eye syndrome was defined as fulfilling any of the following items based on the diagnosis of TAO: ① having one of the subjective symptoms (visual fatigue, discomfort, foreign body, dryness, burning, and visual acuity fluctuation) and Schirmer I test (SIT) ≤ 10 mm; ② One of the subjective symptoms and break-up time (BUT) ≤ 10 s; ③ Positive fluorescein staining of keratoconjunctiva.

Detailed methodology

Evaluating indicator

SIT

The inverted end of the long tear detection filter paper was placed about 5 mm into the outer 1/3rd of the subject's lower eyelid conjunctival sac without ocular surface anesthesia, and the subject was asked to close his eyes gently. After 5 min, the filter paper was taken down, and the SIT value was recorded.

BUT

Corneal topography (Keratograph 5M, type 77000, Germany) was applied. After the subject placed his head in the specified area, the BUT function option of the software, OCULUS Keratograph, was selected. The subject was instructed to look forward without blinking. The value of BUT was obtained after the examination.

Fluorescence staining (FL)

The range of fluorescein staining was observed under the cobalt blue light of the slit lamp microscope after placing the fluorescein staining test paper into the subject’s conjunctival sac of the lower eyelid. The cornea was divided into four quadrants: supratemporal, infratemporal, supranasal, and infranasal. The score was evaluated in every quadrant. The scoring criteria were as follows: 0 for no coloring, 1 for scattered punctate coloring, 2 for diffuse punctate coloring, and 3 for massive coloring. The sum of the scores in each quadrant constituted the corneal FL score with a total of 0–12 points.

Ocular surface disease index (OSDI)

The subjects were required to answer 12 questions, and each question was scored 0–4 points. Nonetheless, the subject could refuse to answer some of the questions. The total score was multiplied by 25 and then divided by the number of questions answered to obtain the final score.

Effective rate

The effective rate was if any of the following three conditions were satisfied. ① The value of SIT was improved by 2 mm after treatment; ② The value of BUT was improved by 2 s after treatment; ③ The FL was decreased by 1 point after treatment.

Follow-up

The clinical data of the subjects were collected at baseline and after 1 month of treatment.

Statistical analysis and blinding

The statistician generated a random sequence, and the clinician who made the evaluation was blind to the grouping. The statistician remained blind until the end of the analysis of the data. The data were statistically analyzed using SPSS 24.0. The continuous data obeying the normal distribution were expressed as mean ± standard deviation, and non-normal data were represented as median (interquartile range). The paired sample t-test or Wilcoxon signed-rank test was used for the comparison between the two groups of paired samples, and the independent sample t-test or Mann–Whitney U test was used for the comparison between the two groups of independent samples. Categorical data were described as the number of cases (percentage), and the chi-square test was used for intergroup comparison. P < 0.05 indicated statistically significant difference.

Problems

Problems anticipated: the follow-up of non Shanghai patients due to the impact of the epidemic

Solutions: Considering the possible loss of follow-up rate as 20%

Safety considerations

The information of all patients should be properly preserved and not disclosed.

Quality assurance

The enrollment information of each patient should be confirmed by at least two clinicians.
